# Multi-MicroRNA Analysis Can Improve the Diagnostic Performance of Mammography in Determining Breast Cancer Risk

**DOI:** 10.1155/2023/9117047

**Published:** 2023-12-27

**Authors:** Ji-Eun Song, Ji Young Jang, Kyung Nam Kang, Ji Soo Jung, Chul Woo Kim, Ah Sol Kim

**Affiliations:** ^1^Department of Family Medicine, Kyungpook National University Chilgok Hospital, 807 Hoguk-ro, Buk-gu, Daegu 41404, Republic of Korea; ^2^BIOINFRA Life Science Inc., Jongno-gu, Seoul 03127, Republic of Korea

## Abstract

The objective of this study was to determine whether multi-microRNA analysis using a combination of four microRNA biomarkers (miR-1246, 202, 21, and 219B) could improve the diagnostic performance of mammography in determining breast cancer risk by age group (under 50 vs. over 50) and distinguish breast cancer from benign breast diseases and other cancers (thyroid, colon, stomach, lung, liver, and cervix cancers). To verify breast cancer classification performance of the four miRNA biomarkers and whether the model providing breast cancer risk score could distinguish between benign breast disease and other cancers, the model was verified using nonlinear support vector machine (SVM) and generalized linear model (GLM) and age and four miRNA qRT-PCR analysis values (dCt) were input to these models. Breast cancer risk scores for each Breast Imaging-Reporting and Data System (BI-RADS) category in multi-microRNA analysis were analyzed to examine the correlation between breast cancer risk scores and mammography categories. We generated two models using two classification algorithms, SVM and GLM, with a combination of four miRNA biomarkers showing high performance and sensitivities of 84.5% and 82.1%, a specificity of 85%, and areas under the curve (AUCs) of 0.967 and 0.965, respectively, which showed consistent performance across all stages of breast cancer and patient ages. The results of this study showed that this multi-microRNA analysis using the four miRNA biomarkers was effective in classifying breast cancer in patients under the age of 50, which is challenging to accurately diagnose. In addition, breast cancer and benign breast diseases can be classified, showing the possibility of helping with diagnosis by mammography. Verification of the performance of the four miRNA biomarkers confirmed that multi-microRNA analysis could be used as a new breast cancer screening aid to improve the accuracy of mammography. However, many factors must be considered for clinical use. Further validation with an appropriate screening population in large clinical trials is required. This trial is registered with (KNUCH 2022-04-036).

## 1. Introduction

Breast cancer continues to disrupt the lives of millions of women. For many years, breast cancer has consistently ranked among the top cancers in women both in terms of incidence and mortality [1]. As early detection is a factor in good prognosis, effective screening strategies are emphasized [2]. The basic options for the primary prevention of breast cancer incidence and mortality are surgical prevention and chemoprevention. Screening is directed at secondary prevention, i.e., not to prevent cancer but to detect it at an early and treatable stage [3–7].

Mammography is a valuable screening and diagnostic tool for breast cancer. Breast cancer mortality has been reduced by mammographic screening. However, mortality has not decreased for women aged 40–59, and screening has a limited impact [8]. These results suggest that the use of mammography should be reevaluated. The number of early stage breast cancer patients has increased since the introduction of mammography, and the number of large (malignant prognosis) tumors detected has decreased proportionally. The statistics may reflect the disproportionate detection of small tumors, some of which may not progress to larger lesions, suggesting an overdiagnosis of breast cancer in some women (cancer found in screening that may not have clinical significance) [9].

The American College of Radiology (ACR) developed a Breast Imaging-Reporting and Data System (BI-RADS) to standardize mammography classifications across the country. Mammograms are now classified according to the BI-RADS classification system for breast reporting [10]. The ACR BI-RADS system includes four breast composition categories: (1) almost entirely fatty, (2) scattered areas of fibroglandular densities, (3) heterogeneously dense, and (4) extremely dense [11].

Other pertinent components of the BI-RADS system include X-ray interpretation by category (0 to 6). Category 0 is “incomplete,” which requires management by recall and needs additional imaging evaluation and/or prior mammograms for comparison. Category 1 is “negative,” which requires management by routine mammography. Category 2 is “benign” and managed by routine mammography. Category 3 is “probably benign,” with a short screening interval (6 months) follow-up or continued surveillance by mammography as management recommendations. Category 4 is “suspicious,” with management recommended by the tissue diagnosis. Category 4 includes 4A (low suspicion for malignancy), 4B (moderate suspicion for malignancy), and 4C (highly suggestive of malignancy). The recommended management of categories 4A, 4B, and 4C is according to the tissue diagnosis. Category 5 is “highly suggestive of malignancy,” with the tissue diagnosis as the recommended management. Category 6 is “known biopsy-proven malignancy,” with surgical excision when clinically appropriate as the management course [11, 12]. Although mammography is the primary screening tool for breast cancer, it is not perfect. The main shortcoming of conventional mammography is a low sensitivity, at approximately 70% [13]. The standard 2-view digital mammogram does not detect all cancers. Its sensitivity decreases as the density of breast tissue increases. In women with dense breast tissue, 76% of cancers are missed [14]. One of the reasons for its limited sensitivity is structural noise created by the overlap of normal breast tissue, which makes it more difficult for a radiologist to perceive cancer that is obscured by normal breast tissue than that not obscured or causes false-positive readings. Therefore, auxiliary tests that can support the limitations of mammography are needed. Ultrasound is usually used as an adjunct to mammography in women with dense breasts and to further evaluate a suspected area. Breast ultrasound has the advantage of detecting small lesions that cannot be detected by mammography and more accurately determining whether the lesion is a hard lesion or a cyst.

However, breast ultrasound has the disadvantage of being incomplete because breast ultrasound alone has a high false-positive rate and the method has difficulty detecting breast microcalcifications [15].

Benign breast diseases are commonly classified as nonproliferative disease, proliferative disease without atypia, and proliferative disease with atypia [16–18]. A high risk of cancer has been observed for all three histological categories. The risk is particularly high for proliferative lesions, especially those with atypia [19–22]. Although the risk is lower for nonproliferative lesions, these lesions account for most benign breast disease diagnoses [19, 21, 23]. Mammographic benign breast diseases are considered strong risk factors for developing breast cancer [19, 24–26]. Women with a history of benign breast disease might have up to a two-fold higher risk of developing breast cancer compared to women without a history of benign breast disease [19, 25]. In addition, benign breast disease and breast density are significantly associated with each other. Forty percent of cancers are contralateral to previous benign breast disease, suggesting that a large proportion of benign lesions might be risk markers rather than precursors of subsequent cancer. Again, there was a strong association between benign breast disease found at screening and the subsequent risk of cancer [25]. This suggests that a highly accurate screening test is needed to differentiate between benign breast disease and breast cancer.

We previously reported an optimal panel of multiple biomarkers and diagnostic models for screening breast cancer in women of all ages. First, we obtained dozens of candidate miRNA biomarkers with differences in expression between healthy control and breast cancer groups using an miRNA microarray and selected nine significant miRNA biomarkers (miR-223, 1246, 206, 24, 373, 21, 6875, 202, and 219B) through a literature search and quantitative real-time polymerase chain reaction (qRT-PCR) analysis. The sample used at this time was used equally for all age groups for both healthy control and breast cancer groups. Although each of the nine miRNAs could be used to screen breast cancer patients, the performance of two or more miRNA combinations improved detection rates. We also analyzed the correlations between the nine candidate miRNA biomarkers using Spearman's correlation analysis and selected a biomarker panel. We decided not to include the highly correlated miRNA together [27].

Based on the results obtained in the first study, we analyzed the performance of two or more combinations of nine miRNA biomarkers and four miRNA biomarkers (miR-1246, 202, 21, and 219B) with the highest performance among those under 50 by age group. In the second study, we compared and analyzed the performance of four miRNA biomarkers (miR-1246, 202, 21, and 219B) in under-50 and over-50 age groups. The set of four miRNA biomarkers was found to be meaningful in the early diagnosis of breast cancer in Korean women under 50 years of age. These four miRNA biomarkers provided higher sensitivity and specificity in patients under 50 years than in all age groups, suggesting that they might be helpful for supplementing mammography sensitivity in Korean women under the age of 50 with a high percentage of dense breasts. Among the four miRNA biomarkers, miR-21 showed significant performance in other carcinomas, whereas the other three miRNAs did not [28].

In this study, we investigated whether the combination of the four miRNA biomarkers (miR-1246, 202, 21, and 219B) could perform uniformly for age groups under 50 and over 50 years. We also tried to confirm the possibility of using these miRNA biomarkers to support mammography results and distinguish benign breast disease from other cancers.

## 2. Materials and Methods

### 2.1. Cohorts and Plasma Samples

This was a retrospective study that used samples and clinical information stored in the Korea Biobank Network that met the research model. It took six months to receive the stored samples. We generated a model from the collected samples and determined breast cancer risk scores for 144 breast cancer patients and 144 healthy control plasma samples. Subjects in the high-risk breast cancer group are more likely to be asymptomatic even if breast cancer occurs. Unlike early breast cancer (stage 0–II), stage III-IV breast cancer is rarely asymptomatic, so patients with this stage were excluded. Those aged 30 to 69 years with a high incidence of breast cancer were selected. The same proportion of samples was collected for each age group in the breast cancer and healthy groups taking into account increases or decreases in biomarker values according to age. We also collected breast cancer samples with BI-RADS categorical information to compare with the performance of mammography currently used clinically for breast cancer screening (Table 1). A model was created and tested with nonlinear algorithm (SVM) and linear algorithm (GLM) analyses to build a breast cancer high-risk group selection algorithm using 60% of the total sample as training data and 40% as validation data (Table 2). Plasma samples from asymptomatic healthy donors and breast cancer patients were obtained from the Korea Regional Biobank of the Korea Institute of Radiological and Medical Sciences. Healthy controls with a known history of cancer, high-grade dysplasia, autoimmune disease, chronic kidney disease, pregnancy, or inflammatory conditions that needed medical management were excluded. Breast cancer samples were obtained before any therapeutic approaches were used. The clinical cancer stage was determined based on the final pathological diagnosis after resection according to the 7th edition of the Union for International Cancer Control tumor-node-metastasis classification. Benign breast cancer disease and other cancer samples were obtained from Inje University Busan Paik Hospital, Korea Regional Biobank of Ajou University Hospital, and the Bio Resources Center of Asan Medical Center (Table 3). This study was ethically approved by the Kyungpook National University Hospital Clinical Trial Review Committee (KNUCH 2022-04-036). Samples were stored at −80°C until analyzed.

### 2.2. Breast Imaging-Reporting and Data System (BI-RADS)

Doctors use a standard system to describe mammography findings and results. When there are multiple findings, the BI-RADS category for the mammography results was assigned the highest category in the following hierarchy, from most to least severe: BI-RADS category for the exam is assigned the highest category in the following hierarchy, from lowest to highest: 1, 2, 3, 6, 0, 4, 5. The vast majority of screening mammograms are categorized as BI-RADS 1 or 2. Screening mammograms with suspicious findings are generally assigned a BI-RADS category of 0 to indicate a callback for diagnostic evaluation, meaning additional views are needed to confirm and further evaluate the findings. In this study, breast cancer samples had BI-RADS categories 3, 4A, 4B, 4C, and 6, which were highly likely to be determined to be breast cancer (Table 1).

### 2.3. Isolation of Circulating RNA from Plasma

Circulating RNA was extracted from 300 *µ*L of plasma using automated nucleic acid extraction equipment (Smart Lab Assist-24; Korea KETT, Seoul, Korea) and finally eluted with 150 *µ*L of RNase-free water. The concentration and purity of the extracted circulating RNA were confirmed using a Thermo Scientific™ NanoDrop™ 2000 spectrophotometer (Thermo Fisher Scientific, Waltham, MA, USA).

### 2.4. Analysis of miRNA Gene Expression with Reverse Transcription and RT-qPCR

qRT-PCR was performed on total RNA using the four miRNAs and internal control (IC) primers for standardization. The reaction solution (total RNA, primers, 2X qRT-PCR master mixture, and ddH_2_O) was added to each of the four prepared tubes. qRT-PCR was performed under the following conditions: 50°C for 15 minutes (1 cycle) ⟶ 95°C for 10 minutes (1 cycle) ⟶ 95°C for 10 seconds, and 65°C for 20 seconds (40 cycles). A Bio-Rad CFX96 Dx system (Bio-Rad, Hercules, CA, USA) was used for genetic analysis. The primer sequences used for PCR were as follows (X in the primer sequence represents inosine): miR-1246 (NR_031648.1) primer sequences, forward 5′-TCT CTXXXT GAA GTA GGA CTG GGC AGA GA-3′ and reverse 5′-CTC AAXXXT GTT TGC AAT AGC CCT TTG AG-3′; miR-202 (NR_030170.1) primer sequences, forward 5′-GGC CAXXXG CAT ATA CTT CTT TGA GGA TCT GGC C-3′ and reverse 5′-CAT GGXXXG ACC GCC CCG TTT TCC CAT G-3′; miR-21 (NR_029493.1) primer sequences, forward 5′-CAGTCXXXG TCG GGT AGC TTA TCA GAC TG-3′ and reverse 5′-CAG TCXXXC AGA CAG CCC ATC GAC TG-3′; miR-219B (NR_039815.1) primer sequences, forward 5′-ACA TCXXXG GAG CTC AGC CAC AGA TGT-3′ and reverse 5′-GTT TGXXXG CGC CAC TGA TTG TCC AAA C-3′; and human hemoglobin subunit beta gene (MK_476504.1) primer sequence, forward 5′-GGA CAX XXC ACT AAG CTC GCT TTC TTG CTG TCC-3′ and reverse 5′-GGA TAX XXG ATG CTC AAG GCC CTT CAT AAT ATC C-3′. Human hemoglobin subunit beta was used as the reference gene for qRT-PCR. The basis for selecting the reference gene was the correlation between RNA concentrations and gene expression (*Ct* value), which is the result of qRT-PCR analysis. The reference gene was most accurate in the *Ct* value range of 25 to 30 and was not different between the healthy control and breast cancer patient groups. The RNA concentrations used in the experiment were standardized to those of the reference gene. Cycle threshold (*Ct*) was defined as the number of cycles required for the fluorescent signal to cross the threshold (i.e., exceeding the background level). Delta (dCt) was defined as *Ct* (reference gene)–*Ct* (target gene).

### 2.5. Statistical Methods, Modeling, and Model Validation

In this study, a classification model was created to distinguish women with breast cancer from normal women using the numerical values of four miRNAs and age information as input variables. The classification method was used for model generation. The classification algorithm used a linear GLM algorithm and a nonlinear SVM algorithm to compare and verify the results. In this study, a similar number of samples were collected from breast cancer patients and normal women according to age in anticipation that the constructed high-risk breast cancer group selection model would yield stable results with no difference in results depending on age group or stage. Additionally, in the case of breast cancer patients, a similar number of patients were collected for each stage. Among 144 breast cancer samples collected and 144 normal women, 84 people, or 60% of the total number of samples collected, were randomly selected to have the same age and stage distributions. Samples not included in the training dataset were automatically selected as verification datasets. The model was generated with the training dataset. The performance of the generated model was comprehensively evaluated using the area under the curve (AUC) value of receiver operating characteristics (ROC) curve analysis. To be specific, a model was built using SVM and GLM methods in a training set consisting of 84 breast cancers and 84 normal women, and 60 breast cancers and 60 normal women were substituted in the validation data to obtain a risk score. ROC curve analysis of risk scores for breast cancer and normal women was used to evaluate the performance of the built model, and the AUC within the ROC was obtained. The closer the area was to 1, the better the performance of the model was.

In addition, the criterion for determining the sensitivity and specificity of the model was set as the value of a normal female sample that satisfied 85% specificity. The sensitivity of the model, its sensitivity and specificity with the verification data, its performance in a benign disease target group, and its performance in a target group of patients with cancers other than breast cancer were evaluated. Statistical analyses in this study were performed using R 4.3.0, a statistical package program used by researchers worldwide that is constantly being developed and modified as the program is shared.

## 3. Results

### 3.1. Modeling and Model Validation Performance Using Nonlinear SVM and Linear GLM Model Analyses

Our previous study confirmed that four miRNA biomarkers (miR-1246, 202, 21, and 219B) performed well in samples of women under the age of 50 and were meaningful for the early diagnosis of breast cancer in Korean women under the age of 50 [28]. The present study reaffirmed the results of previous studies, and the performance of the four miRNA biomarkers was compared with clinical information based on mammography to further confirm the possibility of their ability to support mammographic findings.

Table 2 shows the characteristics of breast cancer patients and healthy control samples used in the training and test sets. We confirmed that all four miRNA biomarkers were meaningful in differentiating normal patients from breast cancer patients. Its performance was observed reliably for all age and stage groups (data not shown).

Two classification algorithms, SVM and GLM, were used to create two models, and their AUC values were found to be high (0.967 and 0.965, respectively). The decision threshold for 85% specificity was determined to be 0.522 for SVM and 0.523 for GLM, with sensitivities of 84.5% and 82.1%, respectively (Table 4). Specifically, when comparing the sensitivity and specificity in women under 50 and over 50, there was a slight difference, but the sensitivity for those under 50 years of age was 100%, demonstrating excellent breast cancer screening performance. The validation dataset, which was independent of the training dataset, was used to assess the performance of the two models. The sensitivity and specificity of the validation dataset were found to be similar for both models (91.7% and 88.3% for SVM and 86.7% and 90% for GLM, respectively). This study also examined differences in the performance of the models according to breast cancer stage. Both models showed consistent performance regardless of breast cancer stage (data not shown).

### 3.2. Performance of Four miRNA Biomarkers in Women under 50 (30–49 Years) and over 50 (50–69 Years of Age)

As a result of breast cancer screening by mammography in Korean women, the breast cancer mortality rate of women aged 50 to 69 has decreased by about 35%. The breast cancer mortality rate has decreased slightly for ages 40 to 49. However, the decrease is statistically insignificant. Women in their 30s to 40s have a high incidence of breast cancer, and those aged 40 to 49 have the highest incidence of breast cancer in Korea [29], suggesting that the value of mammography by age should be reevaluated.

**Figure 1:**
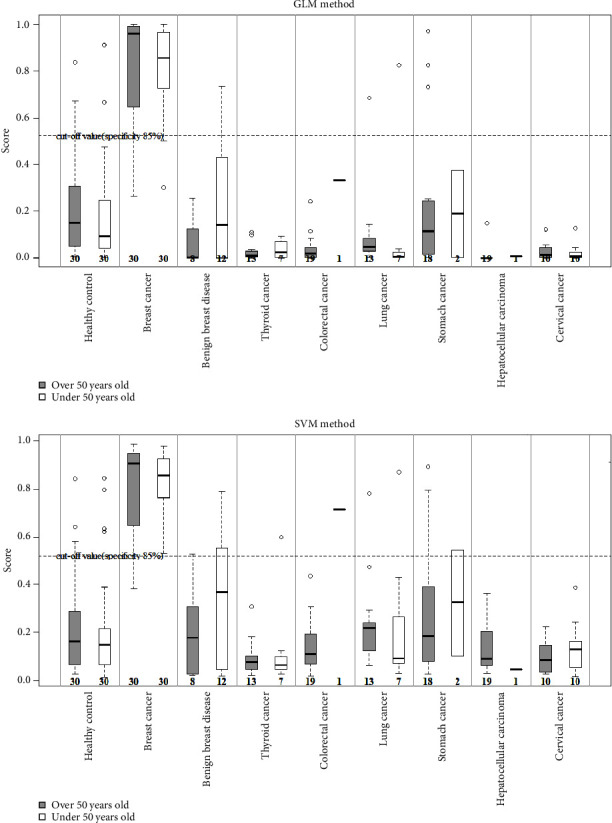
Performance comparison in patients under 50 and over 50 years old. The algorithm for determining the high-risk breast cancer group was set up using the SVM and GLM methods. If the cutoff value was 0.523 or higher, the target was judged to be in the high-risk breast cancer group, and if it was below, it was determined to be in the low-risk breast cancer group. Breast cancer and normal groups were compared in women under 50 years of age (30–49 years) and over 50 years of age (50–69 years), and there was no statistically significant difference by age.

The breast cancer screening performance of the four miRNA biomarkers for patients under 50 (30–49) and over 50 (50–69) was compared. In this study, the distribution of results was analyzed by dividing the verification dataset (60 breast cancer patients and 60 healthy women) into those under 50 and those over 50. The SVM and GLM models showed little difference in breast cancer risk for 30 patients under 50 and 30 healthy women over 50 (Figure 1), and no statistically significant difference was observed in the breast cancer classification performance of women under the age of 50. These results are estimated to show stable breast cancer discrimination performance for all age groups. Thus, multi-microRNA analysis is expected to supplement mammography findings in women under the age of 50.

### 3.3. Comparison of BI-RADS and Multi-MicroRNA Analysis

**Figure 2:**
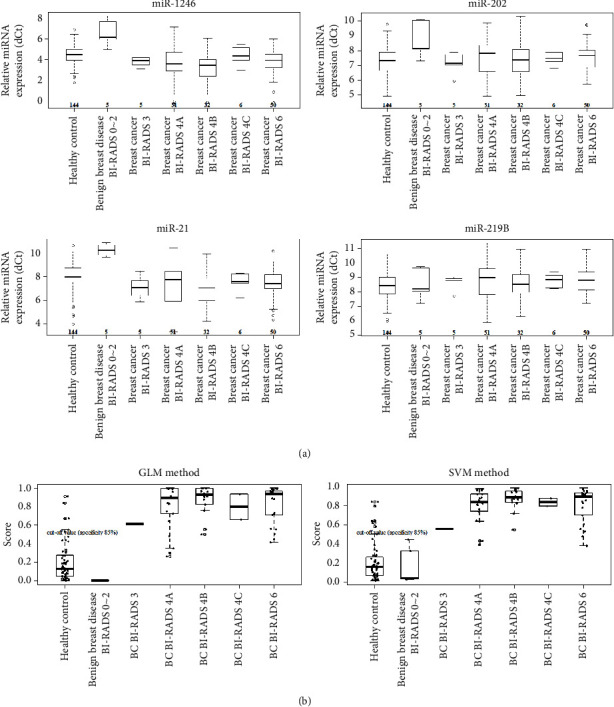
Confirmation of the performance of miRNA biomarkers and algorithms in breast cancer samples in each BI-RADS category. (a) Confirmation of BI-RADS category distribution in breast cancer samples based on the four miRNAs. The expression levels (dCt) of each of the four miRNAs were compared by BI-RADS category. (b) Confirmation of BI-RADS category distribution for the combination of four miRNAs in verification data based on SVM and GLM methodology. The combination of four miRNAs in validation data based on SVM and GLM methodologies was divided into BI-RADS categories and compared based on a high-risk breast cancer cutoff value of 0.523 using verification data based on SVM and GLM methodology (negative controls are samples corresponding to healthy controls and benign breast disease BI-RADS categories 1 and 2).

As mammography has long been regarded as the gold standard for breast cancer screening, it was necessary to determine how well the performance of the four miRNA biomarkers corresponded to mammographic findings. The collected breast cancer patient samples were confirmed by biopsy, and associations with mammographic examination information were determined. The BI-RADS categories of 144 biopsied breast cancer patients were 3 (*n* = 5), 4A (*n* = 51), 4B (*n* = 32), 4C (*n* = 6), and 6 (*n* = 50) (Table 1). For BI-RADS categories 3 and 4A, the biopsy showed a very low probability of breast cancer (less than 10%). Breast cancer risk scores calculated with the four miRNA biomarkers in breast cancer patients did not differ between patients with BI-RADS categories 3, 4A, 4B, 4C, and 6. Thus, these microRNAs are expected to support mammography results for those with BI-RADS categories 3 and 4A. The breast cancer risk score by multi-microRNA analysis was further analyzed by dividing it into BI-RADS categories to investigate the correlation between breast cancer risk score and mammography categories. No significant increase or decrease was observed in the distribution of the expression of the four miRNAs according to the BI-RADS category (3, 4A, 4B, 4C, and 6) in breast cancer samples (Figure 2(a)). Also, no significant increase or decrease was observed according to BI-RADS category distribution for the four miRNAs using validation data based on SVM and GLM methodology (Figure 2(b)). As a specific example, five breast cancer samples with BI-RADS category 3 were used, four of which were included in the training set and used for modeling, one was included in the validation set, and one breast cancer sample with BI-RADS category 3 was correctly determined as being in the high-risk breast cancer group (Figure 2(b)). The analysis based on four miRNAs for both low (BI-RADS categories 3 and 4A) and high breast cancer diagnosis probability categories suggested high breast cancer risk scores for breast cancer patients. However, lower breast cancer risk scores in the analysis of the four miRNAs in non-breast cancers corresponding to BI-RADS categories 3 and 4A require more sample-based comparative analyses to draw accurate conclusions. The results suggest that analyzing the expression of four miRNAs can supplement mammographic evaluations.

### 3.4. Specificity of the Model for Benign Breast Disease and Other Cancers

We determined the specificity of the model in samples with benign breast cancer and other cancers (thyroid, colon, stomach, lung, liver, and cervix) to determine whether breast cancer risk scores could distinguish between benign breast disease and other cancers (Table 5).

**Figure 3:**
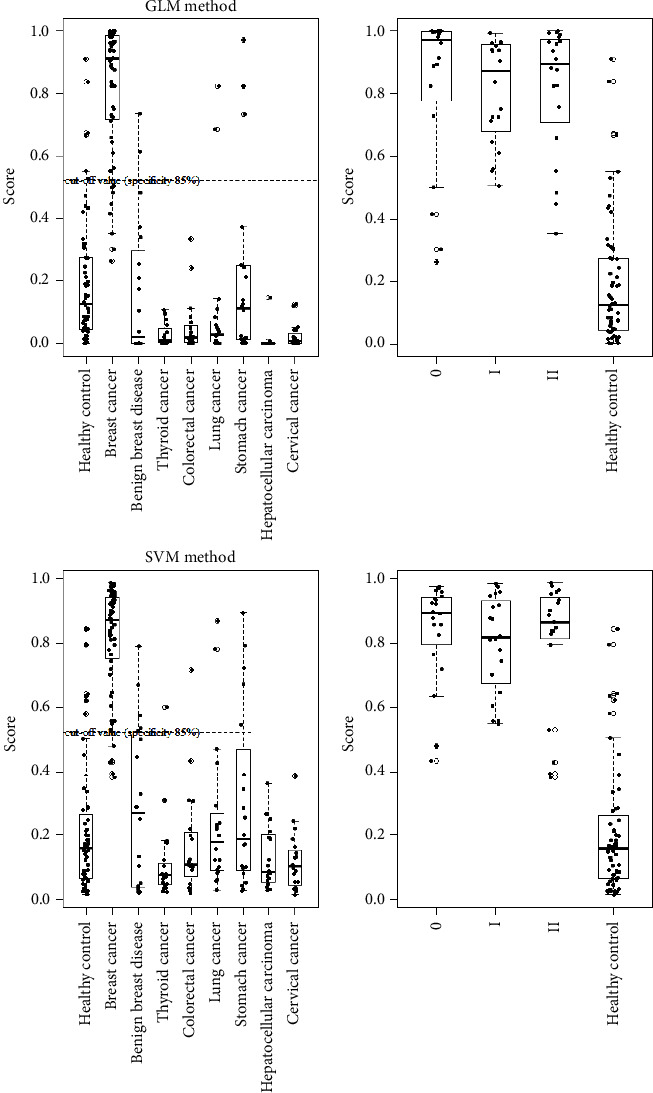
Box plots of the performance of two methods (SVM and GLM). The algorithm for determining the high-risk breast cancer group was established using the SVM and GLM methods. A cutoff value of 0.523 or more was classified into the high-risk breast cancer group, and values less than 0.523 were classified into the low-risk breast cancer group. This was expressed as a box plot. Breast cancer was divided into stages (stages 0, I, and II) and compared to normal groups.

A total of 140 samples, including 20 patients with benign breast tumors and 20 patients each with thyroid cancer, colon cancer, stomach cancer, lung cancer, liver cancer, and cervical cancer, were tested in the SVM and GLM models. The ratio of specificity calculation was calculated when the classification criteria for each classification method were applied to SVM (0.522) and GLM (0.523) and were less than the standard. Both methods showed a high tendency toward correct negative results for benign tumors and cancer types other than breast cancer. In particular, in the results of the GLM model, 90% (18/20) of breast benign tumors were diagnosed as normal. For types of cancers other than breast cancer, the specificity of stomach cancer was the lowest at 85% (18/20), while other types showed a high specificity of 90–100% (Table 5 and Figure 3). These results confirmed that the breast cancer risk calculation model using four miRNA analyses effectively classified benign breast diseases and other cancers. The high specificity for benign breast disease was a very significant result in that it can complement mammographic results.

## 4. Discussion

The incidence rate of breast cancer in Korea and the resulting mortality rate are increasing annually. Breast cancer currently ranks second among female cancers in Korea [29]. Breast cancer is difficult to diagnose early because there are no initial symptoms. However, an accurate, early breast cancer diagnosis is very important because these patients have better treatment results and higher survival rates than those with late detection [30]. Currently, mammography is the only screening test that can lower the mortality rate of patients with breast cancer. However, the sensitivity of mammography is inversely proportional to breast density.

In particular, mammography is less sensitive in women aged 40 to 49 in Korea, where high-density breasts account for more than 70%, than in women aged 50 or older. Thus, breast cancer screening by mammography is not statistically significant [31, 32]. In other words, Korean women have the highest rates of breast cancer in their 30s and 40s and are younger than Western women. The frequency of dense breasts is high in Korean women in their 30s to 40s, limiting the use of mammography for breast cancer screening. Therefore, it is urgent to develop a new breast cancer screening program suitable for women under the age of 50 in Korea.

Korean women have relatively denser breast tissue than Western women. Breast cancer detection is less sensitive in dense breast tissue, and dense breasts have a higher relative risk of breast cancer than fatty breast tissue. Thus, mammographic examination in women with dense breast tissue has limitations, and additional screening images are required in women with dense breast tissue and normal findings [33]. Breast ultrasound is mainly used as an auxiliary test to compensate for the problems of mammography. When ultrasound and mammography are combined, sensitivity is improved compared to mammography alone. However, despite the development of equipment, breast ultrasound is highly dependent on the tester for diagnosis. It is difficult to diagnose breast cancer early due to calcified lesions, and additional tests or biopsies may be needed.

BI-RADS provides a framework for outcome monitoring, as well as recommendations for terminology and final evaluation categories and management for the characteristic analysis of breast lesions [34].

In particular, it is very important to identify malignant diseases as soon as possible among patients with BI-RADS categories 3 and 4A, which are less likely to be malignant. In this respect, this study focused on breast cancer risk based on multi-miRNA analysis of breast cancer patients corresponding to categories 3 and 4A. At the same time, the risk of breast cancer in patients with benign breast disease corresponding to category 3 was compared and analyzed. Although the number of breast cancer patients with BI-RADS category 3 was too small, multi-miRNA analysis could help in diagnosing malignant diseases even if the imaging tests identify BI-RADS category 3. In lesions classified as BI-RADS categories 3 and 4A, malignant disease might be identified in the histopathological results [35, 36]. Multi-miRNA analysis results are expected to help classify subjects who require histopathological examination. For the four miRNA biomarkers to be recognized as an auxiliary test to mammography, the ability to determine malignancy categories 3 and 4A, which are less likely to be malignant, must be secured. However, this study had limitations because the number of samples with BI-RADS category 3 was too small. In subsequent studies, including sufficient samples in each BI-RADS category is critical for an accurate interpretation of the results. In addition, for future integration with existing medical protocols, a prospective research clinical trial is needed to confirm whether the performance of mammography is improved by combining it with mammography currently used in national breast cancer screening.

The results of this study showed that the AUC values of the four miRNA biomarkers (miR-1246, miR-202, miR-21, and miR-219B) measured in plasma for the early diagnosis of breast cancer were 0.967, with a sensitivity of 91.7% and a specificity of 88.3% in the general nonlinear SVM model. In addition, the four miRNA biomarkers were newly confirmed to be effective in distinguishing benign breast diseases from other cancers.

## 5. Conclusion

In conclusion, there was no difference in classification ability between women under the age of 50 (with a high rate of dense breasts, which are difficult to accurately diagnose using mammography) and those over 50 years old using multi-miRNA analysis as a new screening method for high-risk breast cancer. Multi-miRNA analysis could supplement mammographic findings. The value of circulating miRNA as a breast cancer screening diagnostic biomarker has been demonstrated in several previous studies. However, studies comparing miRNA for screening Korean breast cancer patients under the age of 50 and over have not been reported yet. Previous studies have not confirmed whether the developed multi-miRNA sets could distinguish breast cancer from benign breast diseases. However, the present study obtained meaningful results.

Regarding the clinical significance of this study, the results provide a basis for the development of a new adjunct tool to improve the accuracy of mammography. Multi-miRNA analysis can be utilized as a new high-accuracy breast cancer screening tool. Therefore, it is expected that treatment effects and the survival rate of patients with breast cancer can be improved. However, many factors must be considered for its clinical use. Additional validation with an appropriate screening population in large-scale clinical trials is required in the future.

## Figures and Tables

**Table 1:** Characteristics of breast cancer patient and healthy control samples.

Parameter	Healthy control (*n* = 144)	Breast cancer (*n* = 144)
Age (years)	144	144
30∼39	36	36
40∼49	36	36
50∼59	36	36
60∼69	36	36
Stage	—	144
0	—	48
I	—	48
II	—	48
BI-RADS category	—	144
3	—	5
4A	—	51
4B	—	32
4C	—	6
6		50

**Table 2:** Characteristics of breast cancer patient and healthy control samples used in the training and test sets.

Parameter	Training set		Validation set	
	Healthy control	Breast cancer	Healthy control	Breast cancer
Age (years)	Total (*n* = 84)	Total (*n* = 84)	Total (*n* = 60)	Total (*n* = 60)
30∼39	21	21	15	15
40∼49	21	21	15	15
50∼59	21	21	15	15
60∼69	21	21	15	15
Stage	—	Total (*n* = 84)	—	Total (*n* = 60)
0	—	28	N/A	20
I	—	28	N/A	20
II	—	28	N/A	20

**Table 3:** Characteristics of benign breast disease and other cancer patient samples.

Patients	Age (years)	Stage
Benign breast disease (*n* = 20)	30∼69	—
Thyroid cancer (*n* = 20)	30∼69	I∼II
Colorectal cancer (*n* = 20)	30∼69	I∼II
Stomach cancer (*n* = 20)	30∼69	I∼II
Lung cancer (*n* = 20)	30∼69	I∼II
Hepatocellular carcinoma (*n* = 20)	30∼69	I∼II
Cervical cancer (*n* = 20)	30∼69	I∼II

**Table 4:** Modeling and model validation performance using the general nonlinear model (SVM) and linear model (GLM) analysis.

Method	Biomarkers	AUC	Cutoff value	Training set		Validation set					
				Sensitivity (%)	Specificity (%)	Sensitivity (%)			Specificity (%)		
						Total	Under 50	Over 50	Total	Under 50	Over 50
SVM	miR-1246, 202, 21, and 219B	0.967	0.522	84.5	85	91.7	100	83.3	88.3	86.7	90.0
GLM	miR-1246, 202, 21, and 219B	0.965	0.523	82.1	85	86.7	93.3	80.0	90.0	93.3	86.7

**Table 5:** Specificity of benign breast disease and other cancer patients.

Method	Biomarkers	Specificity (%)						
		Benign breast disease (*n* = 20)	Thyroid cancer (*n* = 20)	Colorectal cancer (*n* = 20)	Stomach cancer (*n* = 20)	Lung cancer (*n* = 20)	Hepatocellular carcinoma (*n* = 20)	Cervical cancer (*n* = 20)
SVM	miR-1246, 202, 21, and 219B	75	95	95	75	90	100	100
GLM	miR-1246, 202, 21, and 219B	90	100	100	85	90	100	100

## Data Availability

Deidentifed data can be requested from Dr. Jang at jiyoung.jang@bioinfra.co.kr.
